# Mechanical Performance of Multidirectional Buckling-Based Negative Stiffness Metamaterials: An Analytical and Numerical Study

**DOI:** 10.3390/ma11071078

**Published:** 2018-06-25

**Authors:** Chenhui Ren, Deqing Yang, Haoxing Qin

**Affiliations:** State Key Laboratory of Ocean Engineering, Collaborative Innovation Center for Advanced Ship and Deep-Sea Exploration, School of Naval Architecture, Ocean and Civil Engineering, Shanghai Jiao Tong University, Shanghai 200240, China; renchenhuicn@sjtu.edu.cn (C.R.); qinhaoxing@163.com (H.Q.)

**Keywords:** Buckling-based Negative Stiffness, lattice metamaterial, large deformation, energy absorbing, metamaterial design, advanced manufacturing

## Abstract

Unidirectional, bidirectional and tridirectional Buckling-based Negative Stiffness (BNS) lattice metamaterials are designed by adding prefabricated curved beams into multidimensional rigid frames. Finite Element Analysis models are built, and their mechanical performance is investigated and discussed. First, geometric parameters of the curved beam were systematically studied with numerical analyses and the results were validated by theoretical solutions. Next, within unidirectional designs of different layer numbers, the basic properties of multilayer BNS metamaterials were revealed via quasi-static compressions. Then, the bidirectional and tridirectional designs were loaded on orthogonal axes to research both the quasi-static and dynamic behaviors. For dynamic analysis conditions, simulation scenarios of different impact velocities were implemented and compared. The results demonstrate that the proposed numerical analysis step has accurately predicted the force-displacement relations of both the curved beam and multilayer designs and the relations can be tuned via different geometric parameters. Moreover, the macroscopic performance of the metamaterials is sensitive to the rigidity of supporting frames. The shock force during impact is reduced down below the buckling thresholds of metamaterial designs and sharp impact damage is avoided. The presented metamaterials are able to undergo multiaxial stress conditions while retaining the negative stiffness effect and energy-absorbing nature and possess abundant freedom of parametric design, which is potentially useful in shock and vibration engineering.

## 1. Introduction

Unlike homogeneous natural materials, metamaterials are products of human ingenuity and their properties depend largely on internal construction rather than on parent materials [1]. Over the past few decades, programmed by suitable topology and configuration, these artificial materials have been studied and designed to obtain unconventional characteristics, such as 2D [2,3] and 3D [4,5,6] behaviors of negative or zero Poisson’s ratio, negative or zero compressibility [7,8,9], tunable magnitude and prescribed directionality of thermal expansion [10,11], negative effective mass density and negative effective elastic modulus [12] and low-frequency sound absorption [13].

In the field of mechanical metamaterials, negative stiffness effect has been exploited to obtain innovative properties. Extremely high damping and large stiffness in viscoelastic composites have been realized in previous works of Lakes et al. [14] and Wang et al. [15] with inclusions of negative stiffness. Wang et al. [16] proved that dramatically improved overall properties, such as thermal expansion, piezoelectricity and pyroelectricity, can be achieved in composite materials with an appropriately tuned negative-stiffness phase.

In structures and metamaterials, negative stiffness is usually achieved from beams undergoing both compression and bending [17,18,19], linear springs under rotation [20,21], and specific material distributions [22,23]. With increasing displacement or deformation, these systems experience unloading in the applied force (or provide decreasing resistance). Among these systems, bistable beams are widely used as basic elements in microstructures, and several scholars have been working on them. Vangbo [24] first analyzed the transversal snap-through of a “pre-compressed” slender beam which was loaded at the midpoint. Later Qiu et al. [25,26] presented a bistable mechanism of “pre-fabricated curved beams” that did not rely on compressional residual stress for its bistability. The buckling modal superposition method was used to find analytical expressions of the various midpoint force-displacement relations. Cazottes et al. [27] also investigated a mechanical design of the bistable buckled beam with different actuating force positions.

Based on Qiu’s work, Klatt et al. [28] presented a recoverable and reusable shock-isolating honeycomb structure for absorbing transient mechanical loads, which was designed by periodically repeating a negative stiffness unit cell. Correa et al. [29] fabricated the negative stiffness honeycomb structures using selective laser sintering (SLS) and experimentally evaluated their force-displacement behaviors. The work added rigid central beams to prevent the horizontal expansion during vertical compression and compared the energy-absorption capacity between NS and regular honeycombs. Thereafter a variety of static and dynamic experiments were accomplished for NS honeycombs and the capabilities to mitigate force and absorb energy were studied [30,31,32].

Different lattice metamaterials exhibiting negative stiffness behavior unidirectionally have also been presented by Rafsanjani et al. [33], Restrepo et al. [34], Frenzel et al. [35] and Shan et al. [36]. These metamaterials have all shown excellent mechanical properties, such as rate-independent mechanism, recoverability from large elastic deformation, tremendous freedom of performance-oriented design and extraordinary energy-absorption capacity, especially for dynamic loads. Works done by Goldsberry et al. [37] and Nadkarni et al. [38] have also shown rich dynamic response with distinct regimes of wave propagation and the ability to modify wave propagation in the metamaterial with an external stimulus.

To investigate bistable mechanism in chains, Puglisi et al. [39] studied the hysteretic properties of a discrete chain with bi-stable elements and observed two different kinds of constitutive behavior: hysteretic and stable softening. Benichou et al. [40,41] introduced ideal bi-stable elements, which are connected in series with elastic springs, to investigate protein unfolding and energy releases in muscles. Che et al. [42] used small variations in the unit cell geometry to obtain a deterministic deformation sequence for the unidirectional metamaterial. Findeisen et al. [43] presented a detailed analytical and numerical study of the deformation and energy dissipation of the 3D microlattices in [35], where the unit cell was comprised of buckling beams and hexagonal frames. Liu et al. [44] developed a nonlinear spring-bead model to investigate the dynamic behaviors of phase transforming cellular structures (PTCS) comprised of bi-stable sinusoidal beams.

Experimental and numerical/theoretical researches have been done on multidirectional designs of NS metamaterials. Florijn et al. [45] introduced a soft porous NS metamaterial model which captured programmable mechanics with the help of confinement on one axis. Oh et al. [46] explored an elastic metamaterial including two resonating pairs in its unit cell to adjoin the frequency band of negative density and negative stiffness for various vibration applications. Metamaterial designs showing combined auxetic and bistable properties have also been developed. Inspired by ancient geometric motifs, Rafsanjani et al. [47] presented bistable auxetic metamaterials with square and triangular rotating units. Via parametric studies of geometry profiles, expandability, stiffness and bistability have been proved controllable in the work. The metamaterials designed by Hewage et al. [48] were composed of an auxetic host framework and internal NS elements. To investigate the metamaterial force-displacement relations, four kinds of spring element configurations were compared: conventional springs, PMI foams, buckled beams and magnet assemblies. Several works also introduced 3D metamaterial constructions. The one presented by Duoss et al. [49] has shown distinct negative stiffness effect in shear response. Ren et al. [23] exploited elastic instability to design buckling-induced metamaterials with 3D auxetic behavior, where the unit cell was composed of a solid sphere and three cuboids. The work of Frenzel et al. [35] presented an NS metamaterial block consisting of 3D micro lattices with instability only along the vertical axis. In Shan’s work [36], he also designed 2D and 3D energy-trapping metamaterials, but their mechanical properties were not studied. In addition, topology optimization has been applied to design two-dimensional bistable, compliant periodic structures [50,51]. In brief, all these previous studies contribute to the development of design and manufacturing strategies in negative stiffness metamaterials while only a few of them have presented 2D or 3D microstructural geometry for multiaxial loading conditions.

In the current work, constructions of multidirectional Buckling-based Negative Stiffness (BNS) lattice metamaterials are presented and explored. Under multiaxial loading conditions, the quasi-static force-displacement relations and dynamic characteristics on impact mitigation and energy absorption are investigated. Section 2 presents three different metamaterial designs and describes the foundation of the work, i.e., the behaviors of the curved beams (alone and in unidirectional chains). The mechanical characteristics of the bidirectional and tridirectional BNS metamaterials are researched via numerical simulations in Section 3 and Section 4 respectively, followed by conclusions and some useful design guidelines in Section 5.

## 2. Design of Multidirectional Buckling-based Negative Stiffness Metamaterials

In this section, we extend the concept of BNS Metamaterials to generate multidirectional designs, which are able to undergo multiaxial stress conditions and maintain the negative stiffness. As the constructions of the bi/tridirectional BNS lattice metamaterials stem from the study on the unidirectional case, mechanical behaviors are investigated for the unidirectional design first.

### 2.1. Multidirectional BNS Metamaterial Designs

**Figure 1:**
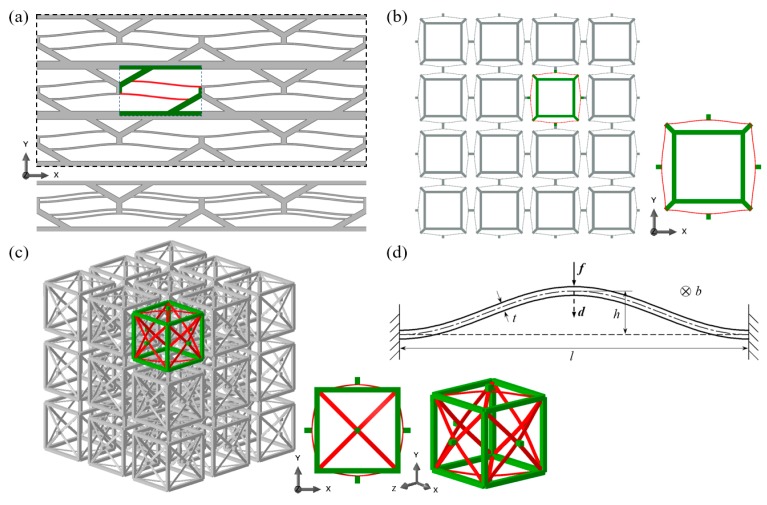
Designs of (**a**) unidirectional, (**b**) bidirectional and (**c**) tridirectional BNS metamaterials and their unit lattices. The supporting frames are plotted in green and the curved beams are in red. (**d**) The centrally loaded curved beam and its geometric parameters. Together with the unidirectional design in (**a**) is another possible configuration with extra curved beams.

The uni/bi/tridirectional BNS metamaterials are illustrated in Figure 1a–c. All the three designs are composed of relatively rigid supporting frames and flexible curved beams. The beams, as depicted in Figure 1d, are the “prefabricated curved beam” investigated in Qiu’s work [26]. The shape is given by *w*(*x*) = *h*/2·[1 ‒ cos(2π*x*/*l*)] where *l* is the beam span and *h* is the initial apex height. Curved beams are assembled row by row toward one, two and three different directions respectively, to ensure that they are loaded laterally at the midpoints, thus negative stiffness effect can be utilized for multiaxial stress conditions. In both the bidirectional and tridirectional designs, adjacent curved beams are connected with short straight rods (See Figure 1b,c). The basic lattice of the bidirectional design is a planar square frame with four oblique beams at its vertices and for the tridirectional design, it is a hexahedral frame with square cross-sections. The investigated bidirectional design contains 4 × 4 lattice units and the tridirectional one contains 3 × 3 × 3 units.

It is worth highlighting that by adjusting their section sizes, the supporting frames can be designed to provide rigid or flexible boundary conditions for the curved beams, resulting in various overall properties of the metamaterials. Meanwhile, to avoid unwanted twisting and rotation at the midpoint and to increase the overall stiffness [26], two or more beams can be clamped together at the center for all the three designs (See Figure 1a). In the current work, for the sake of simplification we set the frames as nearly rigid and have not added any clamped beams into the design. However, in further study on quantitative design of these metamaterials, these issues will be discussed in detail.

**Figure 2:**
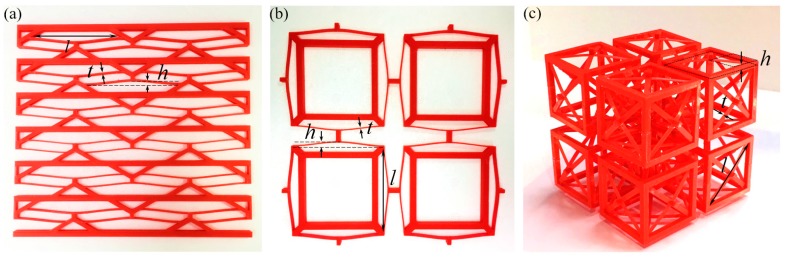
Photographs of the three printed prototypes: (**a**) the unidirectional prototype; (**b**) the bidirectional prototype; and (**c**) the tridirectional prototype.

The main aim of this work is to introduce the metamaterials and preliminarily explore their mechanical performance. Therefore, we set the geometry of the curved beams in all three designs as: *l* = 90 mm, *h* = 4 mm and *t*_bm_ = 1 mm where *t*_bm_ is the beam thickness. The beam depth is 1 mm for the first two designs and 5 mm for the tridirectional one. The values are chosen to ensure that evident negative stiffness can be observed, and no plastic deformation will occur [26,52]. The material parameters used in this work are: *E* = 3283 MPa, *σ*_y_ = 46.6 MPa, *ρ* = 1.21 × 10^−9^ t/mm^3^ and *μ* = 0.38. To show the feasibility of these designs, three prototypes have been fabricated with a Raise3D^®^ Fused Deposition Modeling (FDM) machine and the same polylactic acid (PLA) material, as shown in Figure 2 below. Considering the manufacturing time, only several of the lattices are printed. Some visual demonstrations of the prototypes in the form of short movies can be found in the Supplementary Material.

### 2.2. Parametric Study on the Curved Beam

The theoretical relation between the external force *f* at the midpoint (See Figure 1d) and the transversal displacement *d* can be obtained via a buckling-mode superposition method [26]. The beam deflection *w*(*x*) is expressed as

(1) $$w\left(x\right)=\sum_{i=1}^{\infty }A_{i}w_{i}\left(x\right),$$

where

(2) $$\begin{array}{c} \{\begin{array}{l} w_{i}\left(x\right)=1-\cos\left(n_{i}x\right)\\ n_{i}l=\left(i+1\right)\pi \end{array},\;i=1,3,5,\ldots \;\mathrm{and}\\ \{\begin{array}{l} w_{i}\left(x\right)=1-\cos\left(n_{i}x\right)-\frac{2x}{l}+\frac{2\sin\left(n_{i}x\right)}{n_{i}l}\\ n_{i}l=2.86\pi ,4.92\pi ,6.94\pi ,\ldots \end{array},\;i=2,4,6,\ldots \end{array}$$

Thus, the original as-fabricated shape can be defined as *w*_0_(*x*) = *w*_1_(*x*)·*h*/2.

**Figure 3:**
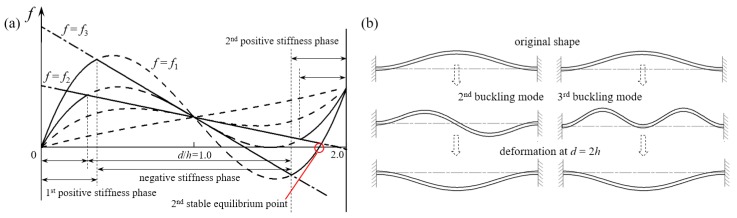
(**a**) Different force-displacement behaviors of the curved beam and (**b**) two potential deforming sequences.

As shown in Figure 3, there are three typical types of *f*-*d* relations during the deformation process (where *d* increases from 0 to 2*h*). At the beginning or the end of the deformation where the axial force is less than the *i*-order buckling threshold, the relation can be defined as *f*_1_ = *f*_1_(*d*). With *d* increasing as well as the axial compression, the axial force will reach and stay at the *i*-order buckling threshold while the bending continues. The force *f* drops down and the *f*-*d* relation can be defined as *f_i_* = *f_i_*(*d*) (*i* takes the value of 2 when the second mode is not constrained, and 3 when the second mode is constrained, and the corresponding deforming sequences are depicted in Figure 3b).

The first type of relation *f*_1_ = *f*_1_(*d*) can be obtained by solving the equation set below:

(3) $$\{\begin{array}{l} A_{1}=\frac{-n_{1}^{2}h}{2\left(n^{2}-n_{1}^{2}\right)}+\frac{4f}{EIln_{1}^{2}\left(n^{2}-n_{1}^{2}\right)}\\ A_{i}=\frac{4f}{EIln_{i}^{2}\left(n^{2}-n_{i}^{2}\right)},\;i=5,9,13\ldots \\ d_{p}=\frac{\pi^{2}h^{2}}{4l}-\sum_{i=1}^{\infty }\frac{A_{i}^{2}{\left(n_{i}l\right)}^{2}}{4l}=\frac{n^{2}t^{2}l}{12}\\ d=h-2\sum_{i=1,5,9\ldots }^{\infty }A_{i}\\ A_{i}=0,i=2,3,4,6,7,8\ldots \end{array}$$

where *E* is the Young’s modulus of the material, *I* is the beam cross-sectional moment of inertia and *d_p_* denotes the axial compression. *n*^2^ = *p*/(*EI*) is introduced here to simplify the expressions and *p* is the axial force. The other two types of force-displacement relations *f*_2_ and *f*_3_ can be deduced by substituting *n* = *n*_2_ and *n* = *n*_3_ into Equation (3) respectively.

As the first three buckling modes play prominent roles in determining the beam deflection, some previous works [29,52] have used them to simplify the superposition and the higher modes were neglected (i.e., *A_i_* = 0, *i* = 5, 9, 13 …). If the higher modes are kept, the third and fourth formula of Equation (3) can be expanded as

(4) $$\sum_{i=1,5,9,13\ldots }\frac{4{\left(n^{2}-n_{1}{}^{2}\right)}^{2}f^{2}}{E^{2}I^{2}n_{i}{}^{2}{\left(n^{2}-n_{i}{}^{2}\right)}^{2}}-\frac{hln_{1}{}^{2}f}{EI}+\frac{n^{2}t^{2}l^{2}{\left(n^{2}-n_{1}{}^{2}\right)}^{2}}{12}-\frac{h^{2}l^{2}n_{1}{}^{2}n^{2}\left(n^{2}-2n_{1}{}^{2}\right)}{16}=0$$

and

(5) $$d=h+\frac{hn_{1}{}^{2}}{n^{2}-n_{i}{}^{2}}-\sum_{i=1,5,9,13\ldots }\frac{8f}{EIln_{i}{}^{2}\left(n^{2}-n_{i}{}^{2}\right)}$$

It is difficult to solve the equation set directly to obtain the *f*-*d* relation, but Equation (4) can be considered as a quadratic equation of unknown *f* if *n* is given as a parameter. To get the *f*-*d* relation, first give a series of discrete values of *n*, then substitute the solutions of Equation (4) into Equation (5) to solve the corresponding displacement *d*.

**Figure 4:**
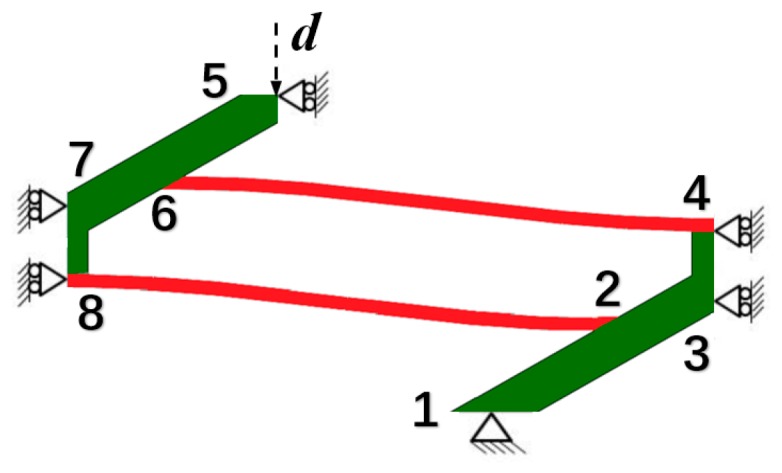
The unit model used in FEA to obtain force-displacement relations of different geometric parameters. The supporting frames are plotted in green and the curved beams are in red. A displacement-controlled load *d* is applied to Joint 5 on the top.

**Figure 5:**
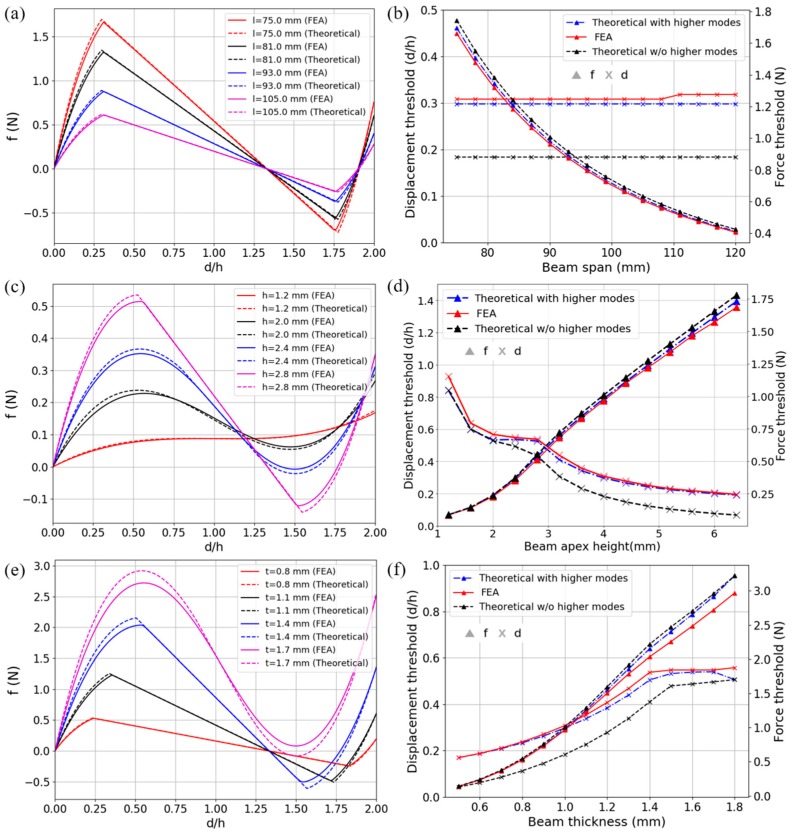
Force-displacement relations of the beams with different geometric parameters. Results from nonlinear FEA and Equations (3)–(5) are compared for different (**a**) span *l*, (**c**) apex height *h*, and (**e**) thickness *t*_bm_. In (**b**,**d**,**f**) are force and displacement thresholds from FEA and theoretical equations (considering or neglecting higher modes). The default parameters are: *l* = 90 mm, *h* = 4 mm and *t*_bm_ = 1 mm, and the varying values are listed in Table 1. The depth is 1 mm for all models.

The results with higher modes have been experimentally validated by Qiu et al. in [26]. Here we exploit Finite Element Analysis (FEA) to enrich the work and explore the *f*-*d* relations of different parameters. Curved beams of different thickness *t*_bm_, span *l,* and apex height *h* are studied. Both the solutions with and without considering higher modes have been given. To obtain the FEA results, a quasi-static implicit analysis step has been utilized for the unit model in Figure 4, taking geometrical nonlinearity into account. In the unit model, two identical half-beams numbered as 6-4 and 8-2 are involved. Joint 1 is fixed and Joints 3, 4, 5, 7 and 8 are only free in vertical translation. A displacement-controlled load, which increases linearly from 0 to 2*h* to ensure a constant loading velocity, is applied to Joint 5 and the vertical reaction force is monitored. Therefore, the force-displacement performance of the unit model should be identical to the theoretical case in Figure 1d, provided the frames are stiff enough to support the beams. The oblique supporting beams make an angle of 30 degrees with the horizontal line and Joints 2 and 6 are the midpoints of Beams 1-3 and 5-7, respectively. Beams 3-4 and 8-7 are perpendicular to the horizontal line and the distance between them is 0.6*l*. The default parameters are set as: *l* = 90 mm, *h* = 4 mm and *t*_bm_ = 1 mm, and only one of them is changed each time. Parameter values are listed in Table 1 below and the final results are depicted in Figure 5. The force threshold and displacement threshold are chosen as key variables, referring to the force value at which the beam starts to buckle and the corresponding transversal displacement, respectively.

As shown in Figure 5, the results from FEA match well with those given by theoretical equations, for both force maximums and displacement thresholds. From Figure 5b,d,f, the results neglecting the higher modes possess relatively accurate force values but cannot capture the displacement thresholds well while the latter is crucial for estimating when the snap-through and snap-back will occur. Other detailed features in Figure 5, such as the trends of the curves for different parameters, are similar to those in Ref. [26] and other works based on it, and thus will not be repeated here. What is noteworthy is that the FEA simulations can conveniently analyze the influence of the frame thickness, which can be another important design parameter. In Figure 5c,e, the deflection occurring on the frames (Beams 1-3 and 5-7 in Figure 4) causes the difference between FEA and theoretical solutions. Besides, although the force maximum is significant for static load bearing and can be enhanced by changing the parameters, it must be carefully chosen for a practical design because the local strain usually increases dramatically at the same time.

### 2.3. Multilayer Behaviors of BNS Metamaterials under Uniaxial Quasi-Static Compression

A finite number of basic units presented in the previous section are connected in series to form a multilayer unidirectional BNS structure and its performance under quasi-static compression is investigated. We choose models for three categories according to different force-displacement behaviors. In the first category, the *f*-*d* curve is monotonic only it includes a horizontal segment, i.e., the stiffness is locally zero. In the second category, the *f*-*d* curve is nonmonotonic and snap-through occurs at the displacement threshold. The minimal force value after buckling is above zero. The last category is similar to the second one except that the minimal force after buckling is below zero, which means it will enter a second stable position (See Figure 3a) when it crosses upward the d-axis again. From the information in Section 2.2, each of these three behaviors can be achieved via different combinations of beam geometric parameters. Here we change one value each time from the standard set *(l* = 90 mm, *h* = 4 mm and *t*_bm_ = 1 mm) to determine the geometric parameters, which are: *h* = 1.2 mm for the quasi-zero monotonic model; *h* = 2.0 mm for the nonmonotonic metastable model; and *l* = 93 mm for the nonmonotonic bistable model.

**Figure 6:**
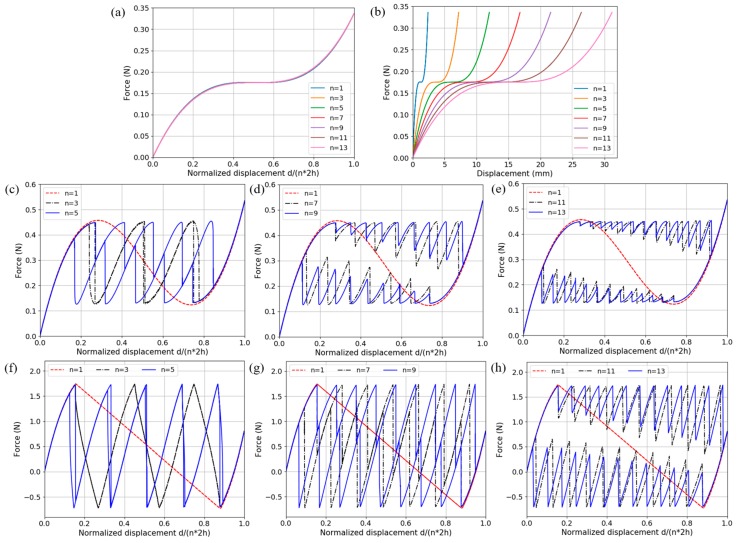
The force-displacement relation diagram of multilayer unidirectional designs. (**a**) Monotonic models with normalized displacement; (**b**) Monotonic models with true displacement; (**c**–**e**) Nonmonotonic metastable models with different layer numbers; (**f**–**h**) Nonmonotonic multistable models with different layer numbers. Parameters are taken as *l* = 90 mm, *h* = 4 mm and *t*_bm_ = 1 mm, except: *h* = 1.2 mm in (**a**,**b**), *h* = 2.0 mm in (**c**–**e**) and *l* = 93 mm in (**f**–**h**).

For all three models, the number of layers increases from 1 to 13 by 2 and similar quasi-static analysis steps are exploited. The displacement-controlled load increases linearly from 0 to *n* × 2*h*, where *n* is the layer number, corresponding to the entire snap-through displacement of all individual layers. Again, the vertical components of displacement and reaction force are monitored. Constraints to restrict lateral expansion are applied to the nodes on two flanks. The results are depicted in Figure 6 below.

The results in Figure 6a,b reveal that the macroscopic *f*-*d* relation does not depend on the number of layers in series, provided the overall displacement is normalized. The structures deform uniformly along the same loading and unloading path. This is because the beam stiffness stays non-negative and no local unloading occurs. In Figure 6b where the displacement is not normalized, the local stiffness decreases when more layers are involved, similar to a series of linear springs. For nonmonotonic *f*-*d* relations shown in Figure 6c–h, there are several common features. First, there are the same amount of snap-through and snap-back events on the loading and unloading paths respectively and that equals to the number of beam layers in series. Second, the force threshold where each layer starts to snap through or snap back keeps constant during the overall deformation, in accordance with the results of the single-beams in Section 2.2. Third, the loading and unloading paths show a tendency of separation with the layer number increasing. Gradually they form a whole cycle and create an enclosed area which refers to the dissipated energy.

In a multilayer nonmonotonic case, the whole model deforms uniformly before one of the layers snaps through at the force threshold. Due to reasons such as numerical error and structural uncertainty, the sequence of layers to buckle is random. (It is noteworthy that the sequence can be controlled by introducing small variations in the unit cell geometry [42].) There will only be one layer snapping through each time since this leads to minimization of the internal strain energy [43]. Once the layer buckles, the overall force on the structure diminishes. The remaining layers will experience unloading and deform back to the original equilibrium point. Since the displacement is controlled, the unloading layers will take the displacement gap induced by the snap-through. This occurs continuously until all the layers deform into the second positive stiffness phase.

The tendency that the overall force reduction (induced by each single-layer buckling) increases with the total compression can be explained using a model of springs in series. When a single layer snaps through, the remaining ones, either in the first or second positive stiffness phase, constitute a system of springs in series. As the stiffness in the second positive stiffness phase is larger than that in the first phase, the stiffness of the whole series raises with more layers in the second phase. Therefore, with a larger stiffness, the system of remaining layers acts more sensitively upon the snap-through, resulting in a larger force reduction. This also explains why the restoring path after each snapping-through becomes steeper and transforms from convex to concave gradually. On the other side, if the number of layers in series increases, the overall force reduction at one snap-through becomes smaller and finally results in the separation between loading and unloading paths. This is because the force reduction is determined by the whole system’s stiffness which decreases with more layers involved. Similar properties of multilayer NS metamaterials have been discussed in [34], where a trilinear analytical model was introduced to describe the constitutive behavior. Therefore, the *f*-*d* curves show a polyline shape. The model is convenient and effective to interpret the multilayer NS behaviors but less satisfactory than FEA in calculating the local stiffness of the system (according to the test results in [34]).

## 3. Performance of the Bidirectional Buckling-based Negative Stiffness Metamaterial

### 3.1. Quasi-Static Compression

**Figure 7:**
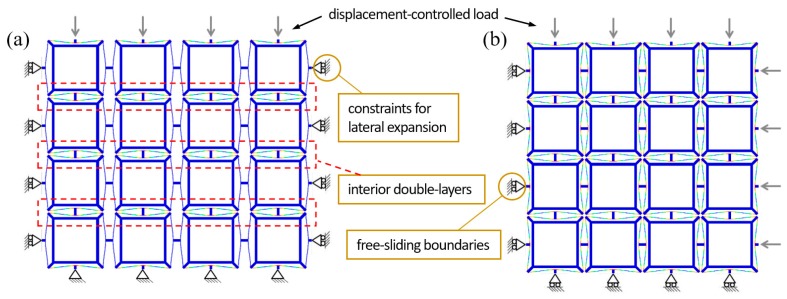
Schematics of boundary conditions and deformation patterns of the bidirectional design under (**a**) uniaxial and (**b**) biaxial compressions.

The quasi-static analysis step in Section 2 is exploited to study the structural performance of the bidirectional BNS metamaterial design under uniaxial and biaxial compressions. As shown in Figure 7, displacement-controlled loads that increase linearly from 0 to *n* × 2*h*, are applied to the exterior nodes facing the investigated directions. Corresponding components of the displacement and reaction force are monitored during the simulations. For the uniaxial compression, two models are added into comparison: with and without constraints to restrict lateral expansion perpendicular to the compression. For the biaxial compression, two displacement-controlled loads increase simultaneously, and corresponding free-sliding boundary conditions are applied on the opposite sides. Figure 7a,b show the deformed configurations at the end of the uniaxial and biaxial compressions, respectively. All the curved-beam layers in the loading directions have snapped through and shaped identical geometrical forms.

**Figure 8:**
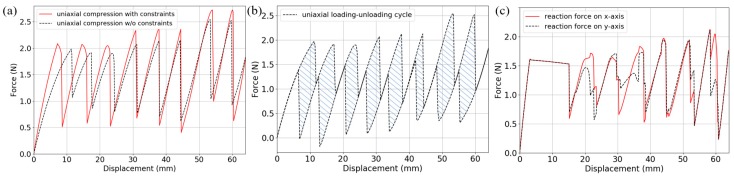
(**a**) Reaction force in the loading direction under uniaxial compression (with and without lateral constraints); (**b**) The loading-unloading cycle under uniaxial compression; (**c**) Reaction force in both directions under biaxial compression.

Figure 8a shows the force-displacement behaviors under the two uniaxial compression conditions introduced above. A typical feature of multilayer BNS structures can be found that the number of snap-through events (abrupt force diminution) on the loading path equals to the number of beam layers in series. In an ideal case, the force thresholds ought to be constant, while in this model they form a stepwise shape. The reasons can be found by observing the deforming animation. The first six peaks correspond to the three interior double-layers (marked in red rectangles in Figure 7a) where the beams are connected to each other by short straight rods. Slight in-plane rotation of these rods occurs during the compression, which causes the twisting of curved beams and decreases the force thresholds. The force threshold of the single beam in this design is about 0.98 N (if the 2nd mode is constrained), so the total force threshold of a layer should be 3.92 N. However, the maximum of these peaks is reduced to 2.72 N due to the twisting and rotation.

Within each double-layer, since the rotation before snapping-through is severer for the first curved beam than the second one, it is not until all the three first-beams snap through (corresponding to the 1st~3rd peaks) that the second-beams start to buckle (corresponding to the 4th~6th peaks). Besides, comparison of the two cases indicates that the constraints have augmented the reaction force by restricting lateral expansion of beam vertices and increasing the strain energy stored in the beams.

The shaded region in Figure 8b represents the energy that is dissipated during the uniaxial loading-unloading cycle. A trapezoidal integration gives the energy values of 46.66 mJ (in total) and 0.95 mJ/g (per unit mass), which are quite small when compared with those (about 60 mJ/g) in Ref. [32]. The reasons can be summarized as: (1) the beams snap through via the 2nd mode, thus the calculated force peaks and valleys are reduced and raised respectively (See Figure 3a); (2) different geometric parameters are used in the two works and the force thresholds (per unit depth) are much larger in [32] (about 2.2 N and 16.5 N, respectively); (3) since the design in this work is for bidirectional load-bearing, the mass of frames takes a larger fraction in the total mass. In general, as the basic mechanisms are the same in these designs, the energy-absorption capacity should be comparative theoretically. A more convincing comparison should be done in future works, which needs further experimental testing and optimal choices of the geometric parameters.

**Figure 9:**
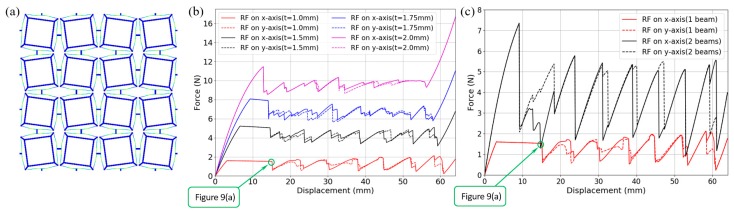
(**a**) Local rotations during biaxial compression; (**b**) Reaction force under biaxial compression (single curved beam with different thickness values); (**c**) Reaction force under biaxial compression (compared with the model which includes two clamped beams). The macroscopic displacement in (**a**) is 15.04 mm which has been marked with green circles in (**b**,**c**).

The *f*-*d* relations in Figure 8c for both axes during biaxial compression share most common characteristics with the uniaxial cases, while possessing a force plateau region right after the incipient positive stiffness part. This region corresponds to the unintentional deformation form in Figure 9a. At the beginning, slight difference and numerical error among the units cause rotation of the short rods and twisting of the curved beams, until the normal deflection in some layers overcomes the twisting with the continuously increasing overall compression. Further analyses with different beam thickness have shown that this phenomenon is not a particular case (See Figure 9b). The force plateau will be shortened when the beams become thicker and the torsional stiffness gets higher, until it finally disappears. As introduced in Section 2, the twisting motion can be eliminated by clamping two or more beams together. In Figure 9c we add the second beam into the model and compare the results (black curves) with the original ones (red curves). The thickness of the second beam has also been set as 1 mm. It can be clearly seen that the force plateau no longer exists.

Similar metamaterial behaviors which arise from broken rotational symmetry, such as the directed buckling of circular pore arrays [45,53] and the buckling of modified regular cellular lattices [23,54], have been reported in the literature. In the presented bidirectional BNS metamaterial design, this has also been revealed as an inherent property. The force plateau region is an important feature and can be elaborately designed in practical applications such as low-speed impact isolation.

Compared with former 2D metamaterials that show simultaneous auxetic and bistable properties [47,48], this bidirectional design lacks the auxetic feature. Under unidirectional compression, the side boundaries will neither expand nor shrink if the frames are sufficiently rigid. On the other hand, although all these designs are tunable in mono/bistability characteristics, only this one is able to exhibit different unstable behaviors in two orthotropic directions. Besides, it avoids the potential problem of stress concentration near compliant hinges and is convenient for integrated manufacturing.

### 3.2. Transient Dynamic Impact

To further study the dynamic behaviors of the proposed BNS metamaterial design, especially the force buffering capacity under impact load, a transient dynamic analysis step has been exploited. Unlike in a static or quasi-static process, the structural response under impact load changes dramatically in a very short period and the effect of inertia cannot be ignored. The transient analysis step simulates a process of two mass blocks impacting the model simultaneously in two orthogonal directions. For each direction, it is similar to the works done by Correa et al. [31] and Qiu et al. [55]. The 1 kg mass blocks are modeled as cubic rigid bodies and their motion is constrained to each impacting direction. Nodes on boundaries (opposite to the blocks) are restrained for normal translation but free of transversal sliding. At the beginning of the analysis step, different initial velocities are directly applied to the blocks to prescribe the input energy and loading rate. The velocities are the same on both axes and increase from 50 to 600 mm/s with intervals of 50 mm/s. Frictionless and separable contact pairs are set between the blocks and the metamaterial model. Reaction force on the support nodes, displacement, velocity and acceleration histories of the mass blocks and the contact force are acquired from the analyses. To control high-frequency oscillations initiated by the buckling, we introduce a small amount of damping. A linear bulk viscosity parameter of 0.12 is taken.

**Figure 10:**
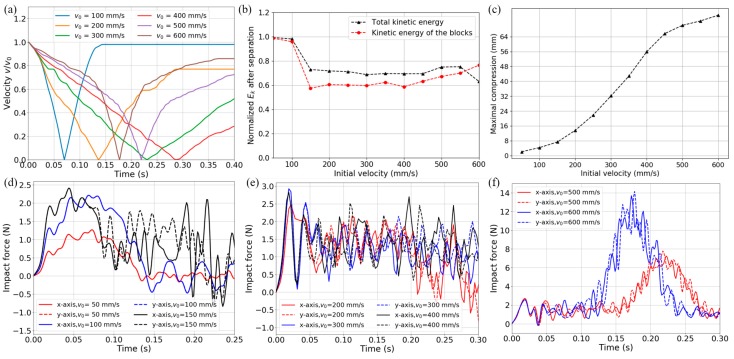
(**a**) Velocity of the block versus time (in the absolute value and normalized by the initial velocity); (**b**) Kinetic energy after the separation (normalized by the input) of different initial impact velocities; (**c**) Maximal compression for different initial impact velocities; (**d**–**f**) Time histories of impact force on two axes for various initial velocities.

Figure 10a depicts the mass block’s velocity-time histories during the impact processes, and they are normalized by the initial values. The minor fluctuations on these curves are caused by overall snap-throughs and local vibration, making the decelerating process strongly nonlinear and more complex than the cases of common cellular structures. We can also clearly observe the velocities changing sign after the zero-speed points. It should be noted that in all cases the velocity finally reaches a constant value if the figure is plotted long enough. Kinetic energy after the separation (when the block leaves the metamaterial and the rebounding velocity rises into the plateau as shown in Figure 10a for *v*_0_ = 100 and 200 mm/s) is normalized by the initial input and given in Figure 10b with regard to different impact velocities. For the velocities of 150~450 mm/s (where snap-throughs occur but without any bottom-outs), the total kinetic energy is about 70% of the initial input and the kinetic energy of the blocks is about 60% of the initial input. In Figure 10c are the distances that the blocks travel until they reach the zero-speed points. It can be observed that the distance increases with the initial velocity, in accordance with more kinematic energy transferred and stored in the metamaterial. Furthermore, the ticks on the vertical axis have been set equal to a single snap-through distance (i.e., 8 mm), thus the number of buckling layers can be found.

Time histories of the reaction force on support nodes are obtained from the simulations and three impact intensity levels are shown in Figure 10d–f, respectively. (I) In Figure 10d the impact velocities are 50, 100 and 150 mm/s and no curved-beam layer buckles during the impact. Therefore, the maximal force value increases with the impact velocity. The whole structure stays in the positive stiffness phase and resists the shock by small deformation. (II) In Figure 10e the impact velocities are 200, 300 and 400 mm/s and different numbers of curved-beam layers buckle according to the velocities. The most important feature of this kind of material/structure can be clearly observed that the maximal force keeps constant below the threshold of a single layer, in spite of the increasing impact velocity and snap-through layer number. (III) In Figure 10f the impact velocities are 500 and 600 mm/s and bottom-outs occur after all the curved-beam layers have snapped through. Even for these situations where the external energy is too large to be transferred and stored inside the metamaterial, the force remains below the threshold value for the first 0.1 s until the occurrence of any bottom-out, which provides a buffer effect for the impact.

## 4. Performance of the Tridirectional Buckling-based Negative Stiffness Metamaterial

### 4.1. Quasi-Static Compression

Similar to Section 3.1, the structural performance of the tridirectional BNS metamaterial design under uniaxial and multiaxial compressions has been studied via quasi-static analysis steps. Displacement-controlled loads are applied to exterior nodes facing the investigated directions and corresponding components of displacement and reaction force are monitored. In the uniaxial compression, two models are added into comparison again: with and without constraints to restrict expansion in the plane perpendicular to the compression. In the biaxial or triaxial compression case, displacement-controlled loads increase simultaneously and linearly from 0 to *n* × 2*h*, while fixed boundary conditions are applied to the opposite sides.

**Figure 11:**
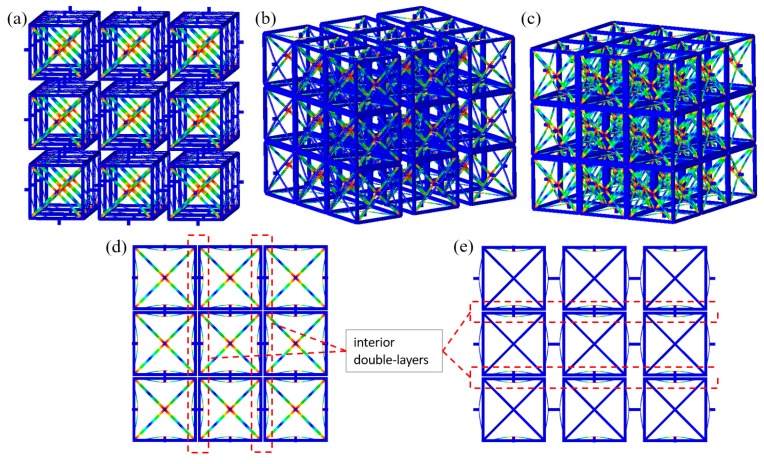
(**a**–**c**) Overall deformation of the tridirectional design under uniaxial, biaxial and triaxial quasi-static compressions; (**d**,**e**) Biaxial and uniaxial in-plane deformations from side views.

Figure 11a–c give the deformed configurations at the end of the uniaxial, biaxial and triaxial compressions, respectively. The in-plane biaxial and uniaxial deformations are depicted in Figure 11d,e. Shaping periodical forms, all the curved-beam layers along the loading directions have snapped through and behaviors on each of the axes show independence of others.

**Figure 12:**
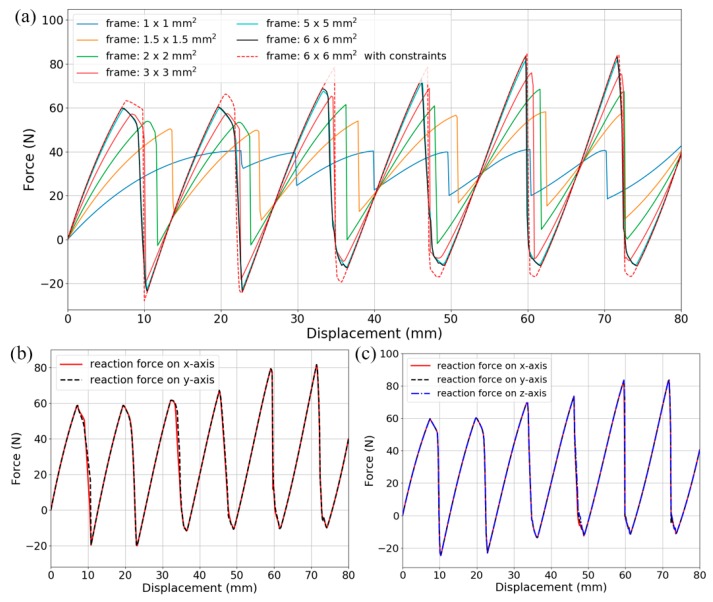
(**a**) Force-displacement relations under uniaxial compression for supporting frames with different cross-sections; (**b**) Reaction force in both directions under biaxial compression; (**c**) Reaction force in three directions under triaxial compression.

The *f*-*d* behaviors under uniaxial, biaxial and triaxial compressions are shown in Figure 12a–c, respectively. Similar to the bidirectional design, the number of snap-through events on the loading path is equal to the number of beam layers in series and the total force threshold corresponds to the sum of the thresholds of beams in parallel. Again, the force thresholds show a stepwise tendency due to the rotation of straight rods connecting the curved beams. Within the two interior double-layers (marked in red rectangles in Figure 11d,e), it is not until all the first-beams snap through (corresponding to the 1st~2nd peaks) that the second-beams start to buckle (corresponding to the 3rd~4th peaks). The 5th~6th peaks refer to the last two exterior beam-layers.

In Figure 12a, supporting frames of different quadrilateral cross-sections are compared and the edge length takes the values of 1, 1.5, 2, 3, 5 and 6 mm. Reducing the cross-sectional area of supporting frames has resulted in the loss of buckling force thresholds and positive load-bearing stiffness (the slope of ascent segments). When the curved beams are pushed at the midpoints, thinner frames stretch more easily. Therefore, more strain energy will be stored inside the frames instead of the curved beams. It can also be found that at 6 × 6 mm^2^, the cross-section of supporting frame is rigid enough and no longer exerts influence on the overall stiffness. The case where lateral constraints are applied still shows slight difference from the “free-boundary conditions” due to the less severe rotation of the connecting rods. In Figure 12b,c, *f*-*d* relations on different axes coincide with each other, indicating the independence of deformation on these axes. Furthermore, no force plateau introduced in Figure 9 was observed here. As shown in the tridirectional design (See Figure 11), the connecting rods are arranged as a *n* × *n* planar array to support the two adjacent lattice-layers, which makes it difficult for the beams to twist. Besides, compared with the bidirectional lattice, two mutually perpendicular beams are clamped together in the tridirectional one (See Figure 1 and Figure 11). Thus, no severe local rotation will take place, nor will the force plateau.

### 4.2. Transient Dynamic Impact

**Figure 13:**
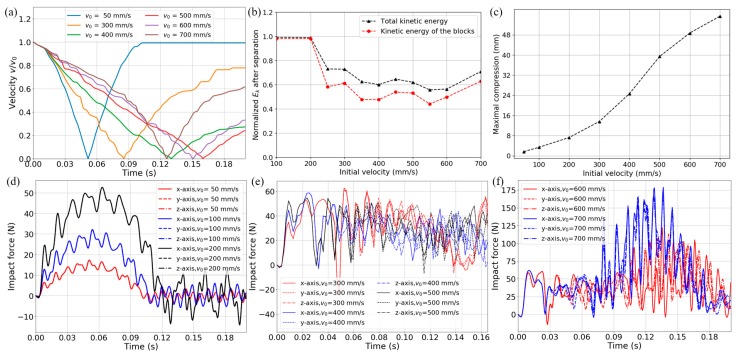
(**a**) Velocity of the block versus time (in the absolute value and normalized by the initial velocity); (**b**) Kinetic energy after the separation (normalized by the input) of different initial impact velocities; (**c**) Maximal compression for different initial impact velocities; (**d**–**f**) Time histories of impact force on three axes for various initial velocities.

Figure 13 shows the dynamic analysis results of the tridirectional metamaterial design. They are obtained from transient steps similar to those in Section 3.2. The metamaterial model is impacted on three orthogonal axes simultaneously by three mass blocks weighing 10 kg and boundary conditions are analogous to the bidirectional case. The impacting velocities are the same on all axes and increase from 50 to 700 mm/s with intervals of 50 or 100 mm/s. Again, reaction force on the support nodes, displacement, velocity and acceleration histories of the mass blocks and the contact force are acquired from the analyses.

The curves in Figure 13 show strong consistency with those of the bidirectional design in Figure 10. Figure 13a depicts the mass block’s normalized *v*-*t* histories. Minor fluctuations, which are caused by overall snap-throughs and local vibration, can be observed on them. The second half of theses curves corresponds to the rebounding of impact blocks. Kinetic energy after the separation (when the block leaves the metamaterial and its velocity rises into a plateau as shown in Figure 13a for *v*_0_ = 50 and 300 mm/s) is normalized by the initial input and given in Figure 13b with regard to different impact velocities. From 350 to 600 mm/s, the total kinetic energy and the kinetic energy of the blocks both show a plateau region, which corresponds to about 60% and 50% of the initial input, respectively. In Figure 13c, the maximal compression increases monotonically, and the ticks on the vertical axis have been set equal to a single snap-through distance, thus the number of buckling layers can be found.

Figure 13d–f demonstrate three intensity levels of the impact force histories. (I) In Figure 13d the impact velocities are 50, 100 and 200 mm/s and no curved-beam layer buckles. (II) In Figure 13e the impact velocities are 300, 400 and 500 mm/s and different numbers of curved-beam layers buckle according to the initial velocities. (III) In Figure 13f the impact velocities are 600 and 700 mm/s and bottom-outs occur after all the curved-beam layers have snapped through. For relatively low impact speed, the whole structure stays in the positive stiffness phase and the deformation is too small to induce any buckling. For moderate impact intensity, different numbers of layers snap through while the maximal force keeps constant below the threshold of a single layer. For high-impact-velocity situations where the external kinematic energy exceeds the absorbing capacity of the structure, bottom-outs will take place along with structural failure. However, the force remains below the threshold before the occurrence of any bottom-out, providing a buffer phase for extreme impacts.

## 5. Conclusions

In this paper, the concept of Buckling-based Negative Stiffness (BNS) lattice metamaterial is extended from unidirectional to bi/tridirectional and FEA models are developed to study their mechanical performance under multiaxial loading conditions. General characteristics and behaviors for both quasi-static and dynamic cases have been preliminarily investigated.

- Geometric parameters of the curved beam are systematically studied in FEA and the results of force-displacement relations show excellent agreement with high-order theoretical solutions. With different parameter combinations, beams of various *f*-*d* behaviors can be designed to form BNS metamaterials and structures. In models comprising multiple curved-beam layers, external energy is absorbed via large elastic deformation while reusability is preserved.
- For the bi/tridirectional metamaterial designs, transient impact simulations show that within the load-bearing capacity, impact energy can be absorbed and stored inside the metamaterial/structure and the reaction force never exceeds the buckling force thresholds. Even for extreme impact situations, the force remains below the thresholds before bottom-outs occur.
- To construct all these BNS metamaterials, relatively rigid supporting frames are added besides the curved beams. They have been proved essential to providing boundary conditions for the beams to exhibit negative stiffness behaviors. Therefore, by specially adjusting their geometry and the corresponding rigidity, the overall performance of the metamaterials can be tuned.
- During the deforming process, if the second mode of the curved beam is not constrained, twisting and rotation at the midpoint may occur and the buckling force threshold will be reduced, which can be favorable or adverse according to different applications. To avoid this from happening, two or more beams can be clamped together at their midpoints.

All these features have shown outstanding design freedom and programmability for BNS metamaterials. The main goal of this paper is to present the configurations of these metamaterials and qualitatively analyze their mechanical properties. The work in the near future will focus on the quantitative design and optimization of the performance. We expect to carry out experimental measurements of the designs and investigate the influence of material properties, such as viscoelasticity. Additionally, the construction from metamaterials to practical structures will be conceived and implemented.

## Figures and Tables

**Table 1:** Geometric parameters used for the systematical research of the curved beam.

Parameter Name	Minimum	Maximum	Interval	Unit
Beam span *l*	75	120	3	mm
Beam apex height *h*	1.2	6.4	0.4	mm
Beam thickness *t*_bm_^1^	0.5	1.8	0.1	mm

^1^ Thickness of the supporting frame *t*_fr_ is set as 6 mm.

## Supplementary Materials

Supplementary Material can be available online at http://www.mdpi.com/1996-1944/11/7/1078/s1.
